# Dietary protein levels changed the hardness of muscle by acting on muscle fiber growth and the metabolism of collagen in sub-adult grass carp (*Ctenopharyngodon idella*)

**DOI:** 10.1186/s40104-022-00747-7

**Published:** 2022-08-25

**Authors:** Min Dong, Lu Zhang, Pei Wu, Lin Feng, Weidan Jiang, Yang Liu, Shengyao Kuang, Shuwei Li, Haifeng Mi, Ling Tang, Xiaoqiu Zhou

**Affiliations:** 1 Animal Nutrition Institute, Sichuan Agricultural University, Chengdu, 611130 China; 2 Fish Nutrition and Safety Production University Key Laboratory of Sichuan Province, Sichuan Agricultural University, Chengdu, 611130 China; 3 Key Laboratory of Animal Disease-Resistance Nutrition, Ministry of Education, Ministry of Agriculture and Rural Affairs, Key Laboratory of Sichuan Province, Sichuan, 611130 China; 4 Healthy Aquaculture Key Laboratory of Sichuan Province, Tongwei Co., Ltd., Chengdu China, Sichuan, 610041 China; 5 Animal Nutrition Institute, Sichuan Academy of Animal Science, Sichuan Animtech Feed Co. Ltd, Chengdu, 610066 China

**Keywords:** Collagen degradation, Collagen synthesis, Grass carp, Muscle fibers, Muscle hardness, Protein

## Abstract

**Background:**

Nutrient regulation has been proven to be an effective way to improve the flesh quality in fish. As a necessary nutrient for fish growth, protein accounts for the highest proportion in the fish diet and is expensive. Although our team found that the effect of protein on the muscle hardness of grass carp was probably related to an increased collagen content, the mechanism for this effect has not been deeply explored. Moreover, few studies have explored the protein requirements of sub-adult grass crap (*Ctenopharyngodon idella*). Therefore, the effects of different dietary protein levels on the growth performance, nutritional value, muscle hardness, muscle fiber growth, collagen metabolism and related molecule expression in grass carp were investigated.

**Methods:**

A total of 450 healthy grass carp (721.16 ± 1.98 g) were selected and assigned randomly to six experimental groups with three replicates each (*n* = 25/replicate), and were fed six diets with 15.91%, 19.39%, 22.10%, 25.59%, 28.53% and 31.42% protein for 60 d.

**Results:**

Appropriate levels of dietary protein increased the feed intake, percentage weight gain, specific growth rate, body composition, unsaturated fatty acid content in muscle, partial free amino acid content in muscle, and muscle hardness of grass carp. These protein levels also increased the muscle fiber density, the frequency of new muscle fibers, the contents of collagen and IGF-1, and the enzyme activities of prolyl 4-hydroxylases and lysyloxidase, and decreased the activity of matrix metalloproteinase-2. At the molecular level, the optimal dietary protein increased collagen type I α1 (*Colα1*), *Colα2*, *PI3K*, *Akt*, *S6K1*, La ribonucleoprotein domain family member 6a (*LARP6a*), *TGF-β1*, *Smad2*, *Smad4*, *Smad3*, tissue inhibitor of metalloproteinase-2, *MyoD*, *Myf5*, *MyoG* and *MyHC* relative mRNA levels. The levels of the myostatin-1 and myostatin-2 genes were downregulated, and the protein expression levels of p-Smad2, Smad2, Smad4, p-Akt, Akt, LARP6 and Smad3 were increased.

**Conclusions:**

The appropriate levels of dietary protein promoted the growth of sub-adult grass carp and improved muscle hardness by promoting the growth of muscle fibers, improving collagen synthesis and depressing collagen degradation. In addition, the dietary protein requirements of sub-adult grass carp were 26.21% and 24.85% according to the quadratic regression analysis of growth performance (SGR) and the muscle hardness (collagen content), respectively.

**Supplementary Information:**

The online version contains supplementary material available at 10.1186/s40104-022-00747-7.

## Introduction

In 1961–2017, the growth rate of total food fish consumption was nearly twice the population growth rate [1]. The expansion of fish consumption was caused not only by the increase in production but also by technological developments in processing methods. According to FAO statistics, the output of grass carp (*Ctenopharyngodon idella*) represented the largest world aquaculture finfish production, accounting for 10.5% in 2018 [1]. However, the processed product of grass carp is scarce because of its poor muscle hardness [2]. In recent years, studies on enhancing the muscle hardness of grass carp have emerged and nutrient regulation has been suggested as an efficient way to improve the muscle hardness of grass carp [3]. Protein is an essential nutrient in the growth of fish and comprises the largest proportion in the fish diet [4]. In our team’s previous research, optimal levels of protein indeed improved the muscle hardness of grass carp [5]. However, the mechanism by which dietary protein improves the hardness of grass carp muscle has not been studied.

In grass carp, muscle hardness is positively correlated with the density of muscle fibers [6]. Muscle fiber density in mammals is associated with the recruitment of new muscle fiber (hyperplasia) regulated by myostatin (MSTN) and myogenic regulatory factors (MRFs) such as MyoD, Myf5 and MyoG [7, 8]. It has been reported that MSTN synthesis in change to: gastrocnemius muscle of male Wistar rats (4 weeks old) was increased by a high protein diet of 35% [9]. Studies demonstrated that moderate levels of dietary protein increased the threonine and glutamate contents of grass carp muscle [5]. Moreover, dietary threonine and glutamate elevated the mRNA levels of *MRFs* in hybrid catfish (*Pelteobagrus vachelli♀* × *Leiocassis longirostris♂*) and Jian carp (*Cyprinus carpio *var. Jian), respectively [10, 11]. These results illustrated that different levels of dietary protein probably affect muscle fiber growth by regulating MSTN and MRFs, which has not been studied and needs further research.

In addition to the density of muscle fibers, the increase in muscle hardness is closely related to the content of collagen [12]. Until now, only few studies have demonstrated that appropriate levels of dietary protein improved the muscle collagen content in grass carp [5], and these studies did not explore the regulatory mechanisms. The content of collagen is mainly determined by its synthesis and degradation. The synthesis of collagen in vertebrates includes the transcription, translation and posttranslational modification, which are regulated by the transforming growth factor-β1 (TGF-β1)/Smads signaling pathway [2], La ribonucleoprotein domain family member 6 (LARP6) [13], prolyl 4-hydroxylases (P4H) and lysyloxidase (LOX) [14, 15]. At present, the effect of dietary protein on collagen synthesis has not been reported. In grass carp, adequate dietary protein levels (25.75%) promoted the expression of the intestinal *TGF-β1* gene [16]. Feeding male Sprague–Dawley rats (3 weeks old) with a 30% high-protein diet increased the concentrations of insulin-like growth factor-1 (IGF-1) in plasma [17] which increased LARP6 and type I collagen expression via PI3K/Akt/P70S6K signaling in human aortic smooth muscle cells [18]. A previous study has already proven that proper levels of dietary protein increased the muscle proline content in grass carp [5]. In large yellow croaker (*Larimichthys crocea*), dietary proline and vitamin C increased the activities of P4H and LOX in muscle [19]. Therefore, dietary protein may affect muscle collagen synthesis, which has not been studied and needs further research. In addition, the degradation of collagen in common carp muscle is mediated by matrix metalloproteinase-2 (MMP-2) [20], and MMP-2 activity is inhibited by tissue inhibitor of matrix metalloproteinase (TIMP) [21]. As mentioned earlier, adequate dietary protein levels increased the expression of the *TGF-β1* gene in the intestines of grass carp. In human hepatic stellate cells, *TGF-β1* inhibited the activity of MMP-2 and upregulated the expression of the *TIMP* gene by activating Smad3 [22]. The above results indicated that dietary protein might regulate muscle collagen degradation, which has not been reported and needs further research.

This study aimed to determine the effect of dietary protein levels on flesh hardness and to explore its regulatory mechanisms by measuring muscle indices related to muscle fiber growth and collagen metabolism, which may provide more theoretical foundation for improving flesh quality by nutritional strategies. In addition, we determined the protein requirements of sub-adult grass carp (*Ctenopharyngodon idella*) according to the growth performance and hardness of muscle, which provides significant direction for aquaculture.

## Materials and methods

### Experimental diet preparation and feeding trial

Healthy sub-adult grass carps (second-year fish [23]) were obtained from a local fishery (Mianyang, Sichuan, China), and no diseases or parasites were observed in the gills, liver or intestines under the microscope according to Fang et al. [24]. After 2 weeks of adaptation, 450 fish were selected (721.16 ± 1.98 g, mean ± SD) and assigned randomly to 18 experimental floating net cages (6 treatments), resulting in 3 cages per treatment and 25 fish per cage (140 cm × 140 cm × 140 cm). Each cage was equipped with a disc of 80 cm in diameter to collect the uneaten feed as reported by Wu et al. [25]. The formulation and approximate composition of the six diets with graded protein levels of 16%, 19%, 22%, 25%, 28% and 31% are presented in Table 1 and the actual protein contents of diets were determined as 15.91%, 19.39%, 22.10%, 25.59%, 28.53%, and 31.42%. As a source of dietary protein, the fish meal:casein:gelatin ratio was 13:31:6 as reported by Xu et al. [16]. The amino acid pattern was simulated using crystalline amino acids [26]. Furthermore, the six diets were designed to be iso-energetic (digestive energy: 13.89 kJ/g diet) and iso-lipidic (4.7%) according to Wang et al. [27]. After all the ingredients were fully mixed, water was added, and the mixture was pelleted through a screw extruder, air-dried as reported by Jiang et al. [28] and stored at 4 °C. For the 60 d management, fish were fed the corresponding experimental diets four times daily to satiation. After feeding for 30 min, the uneaten feed on the disc was dried and weighed to calculate the feed intake as described by Cai et al. [29]. During the feeding trial, river water was pumped through sand filters for purification and was flowed into each cage. The maintenance water was changed every week, and micropore aeration was maintained by an oxygen autosupplemention system as reported by Wu et al. [25]. Under natural weather and light conditions, the water temperature, pH and dissolved oxygen were maintained at 23.9 ± 3.3 ℃, 7.5 ± 0.5, and no less than 6.0 mg/L, respectively.

**Table 1:** The formulation and approximate composition analysis of the six diets

Ingredients, %	Dietary protein levels, %					
	15.91	19.39	22.10	25.59	28.53	31.42
Fish meal	5.40	6.43	7.46	8.48	9.51	10.54
Casein	12.89	15.34	17.78	20.23	22.68	25.12
Gelatin	2.50	2.97	3.44	3.92	4.39	4.86
α-starch	18.00	18.00	18.00	18.00	18.00	18.00
Corn starch	44.60	41.11	37.61	34.13	30.60	27.10
Microcrystalline cellulose	6.68	6.34	6.00	5.64	5.33	5.00
Fish oil	2.43	2.34	2.26	2.17	2.09	2.00
Soybean oil	1.76	1.76	1.76	1.76	1.76	1.76
Vitamin premix^a^	1.00	1.00	1.00	1.00	1.00	1.00
Mineral premix^b^	2.00	2.00	2.00	2.00	2.00	2.00
Ca(H_2_PO_4_)_2_	1.67	1.64	1.61	1.59	1.56	1.53
Choline chloride premix^c^	1.00	1.00	1.00	1.00	1.00	1.00
Ethoxyquin	0.05	0.05	0.05	0.05	0.05	0.05
*L*-Trp	0.03	0.03	0.03	0.04	0.04	0.05
Nutrient contents, %						
Protein	16.00	19.00	22.00	25.00	28.00	31.00
Lipid	4.70	4.70	4.71	4.71	4.72	4.72
n-3	1.02	1.02	1.02	1.02	1.02	1.02
n-6	0.94	0.94	0.94	0.94	0.94	0.94
Digestive energy, KJ/g	13.89	13.89	13.89	13.89	13.89	13.89

^a^Per kilogram of vitamin premix (g/kg): retinyl acetate (500,000 IU/g), 0.19; cholecalciferol (500,000 IU/g), 0.20; *DL*-α-tocopherol acetate (50%), 23.2; menadione (50%), 0.38; cyanocobalamin (1%), 0.94; *D*-biotin (2%), 0.75; folic acid (95%), 0.17; thiamine nitrate (98%), 0.09; ascorhyl acetate (95%), 9.77; niacin (99%), 3.44; meso-inositol (98%), 28.5; calcium-*D*-pantothenate (98%), 3.85; riboflavin (80%), 0.73; pyridoxine hydrochloride (98%), 0.45. All ingredients were diluted with corn starch to 1 kg

^b^Per kilogram of mineral premix (g/kg): MnSO_4_·H_2_O (31.8% Mn), 2.66; MgSO_4_⋅H_2_O (15.0% Mg), 256.79; FeSO_4_·H_2_O (30.0% Fe), 12.61; ZnSO_4_·H_2_O (34.5% Zn), 8.87; CuSO_4_.5H_2_O (25.1% Cu), 0.95; Ca (IO_3_)_2_ (3.2% Ca), 0.0668 g; Na_2_SeO_3_ (45% Se), 6.07. All ingredients were diluted with corn starch to 1 kg

^c^Per kilogram of choline chloride premix contains choline chloride (50%) 261.95 g, the rest diluted with corn starch to 1 kg

### Sample collection and biochemical analysis

At the termination of the feeding trial, fish in each treatment were counted and weighed after anesthesia in a benzocaine bath (50 mg/L), and then the percentage of weight gain (PWG) and specific growth rate (SGR) were calculated. After that, nine of the fish were selected randomly and euthanized by cervical puncture as described by Montenegro et al. [30]. The left muscle of the fish was immediately removed on ice as described by Ma et al. [31]. A part of the muscle was used to measure pH value by using a calibrated pH probe (Testo 205 pH meter, Testo AG, Lenzkirch, Germany). The second part of the muscle was used to determine the cooking loss and shear force according to the methods of Adeyemi et al. [32]. The rest of the muscle was stored at –80 ℃ after rapid freezing in liquid nitrogen for later analysis. A piece of the right muscle was fixed in 4% paraformaldehyde solution for later morphological analysis.

The moisture, protein, and lipid contents in grass carp muscle were determined by oven drying, the Kjeldahl method, and the Soxhlet exhaustive extraction technique respectively as reported by Horwitz et al. [33]. The free amino acid content of muscle was determined using an L-8900 amino acid analyzer (Hitachi, Ltd., Tokyo, Japan). The fatty acid profile was determined using gas chromatography. Lipids were extracted using a chloroform–methanol mixture (2:1) from 200 mg of freeze-dried muscle powder. After saponification with 0.5 mol/L methanolic potassium hydroxide as described by Yu et al. [34], fatty acid methyl esters were obtained using 14% boron trifluoride-methanol solution to methylate the fatty acids as described by Shantha et al. [35]. The samples were loaded in a GC-2010Plus (Shimadzu, Co., Ltd., Kyoto, Japan) equipped with an SP-2560 (100 m × 0.25 mm I.D. and 0.2 μm film thickness) capillary column and flame ionization detector. The detector and injector temperatures were 250 ℃ as described by Montenegro et al. [36]. The column temperature program was as follow: 5 min at 140 °C, followed by a temperature increase of 8 °C/min to 200 °C, from 200 °C to 220 °C at 2 °C/min, from 220 °C to 240 °C at 5 °C/min. Nitrogen was used as carrier gas and was kept at a rate of 1.8 mL/min. The injected sample volume was 1 μL. A 37-fatty acid methyl ester mix (Sigma-Aldrich, St. Louis, MO, USA) was used as the external standards, and the results were expressed as a percentage of total fatty acids.

5′-Inosinic acid (IMP, CJ-F01630) was measured using an ELISA test kit (Shanghai Changjin Biotechnology Co., Ltd, Shanghai, China). The contents of hydroxyproline (HYP, A030-2-1) and lactate (A019-2-1) were measured using test kits (Nanjing Jiancheng Bioengineering Institute, Nanjing, Jiangsu, China). The collagen content was calculated by multiplying the Hyp content by eight according to AOAC [37]. The IGF-1 content (ML790132), and the activities of P4H (YJ760958), LOX (YJ790382) and MMP-2 (ML7101146) were analyzed using ELISA test kits (Shanghai Enzyme-linked Biotechnology Co., Ltd, Shanghai, China).

### Morphometric analysis

After serial dehydration in gradually increasing concentrations of ethanol, the muscle tissues were equilibrated in xylene and embedded in paraffin. The samples were dissected into 3–5 μm slides, dyed with H&E and observed using a Nikon TS100 light microscope (Nikon, Kyoto, Japan). Morphometric analysis, including myofiber density and frequency of different diameter muscle fibers, was performed using Image-Pro Plus® 4.5 image analysis software. The fiber density was calculated based on the number of fibers per mm^2^ of muscle cross-sectional area according to the method of Johnston et al. [38].

### Quantitative real-time PCR analysis

Total RNA from fish muscle samples was isolated by an RNAiso Plus kit (9108Q, Takara Biotech Co., Ltd., Tokyo, Japan). The purity and quantity of RNA were identified via agarose gel (1.5%) electrophoresis and NanoDrop 2000 (Thermo Fisher Scientific, Waltham, MA, USA), respectively. Then, the RNA was reverse transcribed to cDNA by a PrimeScript® RT reagent kit (RR047A, Takara, Tokyo, Japan). Quantitative real-time PCR was performed by specific primers designed according to the sequences of grass carp (Additional file 1: Table S1). β-Actin was chosen as an internal reference gene for normalization according to our pre-experiment results. The results were analyzed according to the 2^−ΔΔCT^ method.

### Western blotting

Primary antibodies against total-Smad2 (AF6449, 1:1000), p-Smad2 (AF3449, 1:1000, Ser467), Smad4 (AF5247, 1:1000), total-Akt (AF6261, 1:1000), and p-Akt (AF0016, 1:1000, Ser473) were purchased from Affinity BioReagents (Golden, Colo, USA). Anti-Smad3 (A11471, 1:1000) and LARP6 (A0008, 1:1000) were purchased from ABclonal Technology (Wuhan, Hubei, China). The control protein used was β-actin (AF7018, 1:1000, Affinity). These antibodies were checked and could successfully cross-react with grass carp proteins of interest.

The procedure for muscle protein extraction was performed using lysis buffer (P0013B, Beyotime, Shanghai, China). After extraction, the total protein concentrations were determined using the Bio-Rad protein assay kit (5000001, Bio-Rad, Hercules, CA, USA). The target protein was separated by SDS-PAGE and then transferred to a PVDF membrane for WB analysis. The membrane was blocked with blocking solution (0.5%) for 1.5 h at room temperature and then incubated with primary antibody overnight at 4 °C. Then, the membranes were washed and incubated with goat anti-rabbit horseradish peroxidase-conjugated secondary antibody (A0208, 1:8000, Beyotime) for 2 h. Finally, the immune complexes were visualized with an ECL kit (KF8001, Affinity) under ChemiDoc imaging system (Bio-Rad, Hercules, CA, USA). The relative amount of target proteins was analyzed by Image Lab 6.1 software. This experiment was repeated six times.

### Calculation and statistical analyses

The calculation formula is as follows:

$$\mathrm{Percentage}\;\mathrm{weight}\;\mathrm{gain}\;(\mathrm{PWG},\;\%)\:=\:100\:\times\:\lbrack\mathrm{FBW}\;(\mathrm g/\mathrm{fish})-\mathrm{IBW}\;(\mathrm g/\mathrm{fish})\rbrack/\mathrm{IBW}\;(\mathrm g/\mathrm{fish})$$

- $$\mathrm{Specific}\;\mathrm{growth}\;\mathrm{rate}\;(\mathrm{SGR},\;\%/\mathrm d)\:=\:100\:\times\:\lbrack\ln\;(\mathrm{mean}\;\mathrm{final}\;\mathrm{weight})-\ln\;(\mathrm{mean}\;\mathrm{initial}\;\mathrm{weight})\rbrack/\mathrm{days}$$
  $$\mathrm{Feed}\;\mathrm{efficiency}\;(\mathrm{FE},\;\%)\:=\:100\:\times\:\mathrm{WG}/\mathrm{FI}$$

Data are presented as the mean ± SD, and were subjected to one-way analysis of variance (ANOVA) and Duncan’s multiple-range test to check for significant differences among the treatment groups at *P* < 0.05. Before statistical analysis, all data were tested for homogeneity of variance and normal distribution by using Levene’s test and Shapiro–Wilk test, respectively (SPSS 21). The linear and quadratic effects of increasing the dietary protein level were conducted by orthogonal polynomial contrasts which was performed by using PROC MIXED (SAS software version 9.4) as described by Lu et al. [39]. The correlation analysis between indicators was calculated using Pearson’s correlation coefficient (SPSS 21). To determine the appropriate dietary protein levels, we used a quadratic regression model for estimation referring to Zhao et al. [40].

## Results

### Growth performance

Growth performance is shown in Table 2. After 60 d of feeding, the FBW, PWG and SGR significantly increased with dietary protein levels rising to 22.10% (*P* < 0.05) and then showed no significant difference in higher protein level groups. The FI was significantly increased as dietary protein rose to 22.10% (*P* < 0.05) and gradually decreased thereafter. The FBW, PWG, SGR and FI showed significant quadratic regression with dietary protein levels (*P* < 0.05). The FE was the lowest in the dietary protein level 15.91% and the maximum value appeared in the 31.42% group. There was a significant linear regression relationship between FE and dietary protein levels.

**Table 2:** The growth performance of grass carp fed with different dietary protein

Items	Dietary protein levels						Linear		Quadratic	
	15.91%	19.39%	22.10%	25.59%	28.53%	31.42%	*F*	*P*	*F*	*P*
IBW, g	721.60 ± 1.60	720.80 ± 1.60	722.13 ± 1.85	720.00 ± 2.12	721.87 ± 3.03	720.53 ± 2.44	0.17	0.69	0.00	0.96
FBW, g	1176.00 ± 34.06^a^	1233.99 ± 108.08^ab^	1381.60 ± 56.30^c^	1333.60 ± 96.64^bc^	1327.02 ± 60.66^bc^	1331.94 ± 27.76^bc^	8.82	< 0.05	5.59	< 0.05
PWG, %	62.98 ± 4.94^a^	71.20 ± 15.13^ab^	91.34 ± 8.27^c^	85.23 ± 13.50^bc^	83.81 ± 7.65^bc^	84.85 ± 3.26^bc^	8.93	< 0.05	5.58	< 0.05
SGR, %/d	0.81 ± 0.05^a^	0.89 ± 0.15^ab^	1.08 ± 0.07^c^	1.02 ± 0.12^bc^	1.01 ± 0.07^bc^	1.02 ± 0.03^bc^	9.38	< 0.01	5.46	< 0.05
FI, g	993.85 ± 0.60^a^	1043.83 ± 2.83^c^	1233.27 ± 1.01^f^	1141.44 ± 1.22^e^	1065.50 ± 1.72^d^	1004.09 ± 0.40^b^	10.91	< 0.01	40,066.6	< 0.01
FE, %	45.72 ± 3.52^a^	49.18 ± 10.50^ab^	53.47 ± 4.66^ab^	53.75 ± 8.46^ab^	56.80 ± 5.47^ab^	60.89 ± 2.56^b^	9.99	< 0.01	0.00	0.96

Values are means ± SD and *n* = 3 for each group. ^a-f^ Different superscripts in the same row indicate significantly difference (*P* < 0.05)

### Effects of dietary protein on nutrient contents in the muscle

The nutrient contents of muscle are shown in Table 3. The moisture content of muscle was reduced with dietary protein rising to 22.10% and then had no apparent difference at higher protein levels (*P* < 0.06). The maximum muscle crude protein and lipid contents were observed at protein levels of 25.59% and 22.10%, respectively, and were reduced thereafter (*P* < 0.05). The cooking loss was enhanced with the increase in dietary protein. The pH values in the muscle were the lowest in the 15.91% and 31.42% protein groups, while the lactate content was the highest in these two diets. The moisture and crude lipid contents showed significant quadratic regression with dietary protein levels (*P* < 0.05). The cooking loss, contents of crude protein and lactate showed significant linear and quadratic regression relationship with dietary protein levels (*P* < 0.05).

**Table 3:** The nutrition composition and physicochemical parameter in the muscle of grass carp

Items	Dietary protein levels						Linear		Quadratic	
	15.91%	19.39%	22.10%	25.59%	28.53%	31.42%	*F*	*P*	*F*	*P*
Moisture, %	78.99 ± 2.30^b^	76.44 ± 0.53^ab^	75.46 ± 0.96^a^	75.35 ± 1.29^a^	76.90 ± 4.95^ab^	76.34 ± 0.75^ab^	2.22	0.15	5.20	< 0.05
Crude protein, %	15.38 ± 1.50^a^	18.46 ± 0.69^bc^	19.36 ± 1.04^c^	20.83 ± 0.91^d^	19.02 ± 1.78^bc^	17.84 ± 0.34^b^	15.51	< 0.01	56.16	< 0.01
Crude lipid, %	3.51 ± 0.10^a^	3.72 ± 0.16^ab^	4.03 ± 0.16^c^	3.93 ± 0.39^bc^	3.72 ± 0.32^ab^	3.71 ± 0.14^ab^	1.25	0.27	12.81	< 0.01
Cooking loss, %	22.78 ± 1.67^a^	23.11 ± 0.64^a^	23.00 ± 0.68^a^	24.72 ± 0.74^b^	25.04 ± 0.44^b^	26.86 ± 0.53^c^	85.30	< 0.01	7.66	< 0.01
pH_24h_	6.46 ± 0.03^ab^	6.52 ± 0.05^ab^	6.49 ± 0.07^ab^	6.50 ± 0.14^ab^	6.53 ± 0.02^b^	6.41 ± 0.12^a^	0.38	0.54	3.68	0.06
Lactate, mmol/g prot	3.72 ± 0.26^b^	2.83 ± 0.36^a^	2.79 ± 0.32^a^	2.92 ± 0.19^a^	2.96 ± 0.21^a^	3.00 ± 0.10^a^	12.24	< 0.01	26.55	< 0.01

Values are means ± SD and *n* = 6 for each group. ^a-d^ Different superscripts in the same row indicate significantly difference (*P* < 0.05)

Table 4 shows the fatty acid profile in grass carp muscle. The ∑SFA apparently reduced (*P* < 0.05) with dietary protein increasing, and gradually increased thereafter. The ∑UFA and ∑MUFA were obviously greater at dietary protein level 22.10% compared with that in 15.91% and 31.42% groups (*P* < 0.05), while the ∑PUFA was opposite. The contents of ∑SFA, ∑UFA and ∑MUFA were observed to be significant linear and quadratic affect by dietary protein levels (*P* < 0.05). The contents of C14:0, C16:0, C18:0, C14:1, C18:2n6c, C20:2 and C20:3n3 of grass carp muscle showed no obvious differences among all groups (*P* > 0.05). With the increase of dietary protein, the C15:0 content increased (*P* < 0.05). The C16:1 content was the highest when the dietary protein was 19.39%. The C17:0 content was increased with the increase in dietary protein from 15.91% to 25.59% (*P* < 0.05), then plateaued. The C17:1 content increased gradually as dietary protein rose to 25.59% and decreased thereafter (*P* < 0.05). The maximum of C18:1n9t content appeared in the 31.42% protein group and there were no apparent differences when dietary protein was less than 31.42%. The contents of C20:0, C18:1n9c, C20:1n9, C18:3n3, and C18:3n6 were remarkably increased with the dietary protein increasing from 15.91% to 22.10% (*P* < 0.05), and plateaued thereafter. The C22:0 content was the highest in the 15.91% dietary protein level (*P* < 0.05). The contents of C23:0, C20:5n3, C22:6n3 were remarkably higher in the 15.91% protein group compared to 19.39%–31.42% protein groups (*P* < 0.05). The C22:2 content showed no significant difference when dietary protein levels were 15.91%–28.53%, and the lowest content was observed at the 31.42% dietary protein (*P* < 0.05). The contents of C15:0, C16:1, C17:0, C17:1, C18:1n9t, C22:2 and C20:5n30 were observed to have a significant linear relationship with dietary protein levels (*P* < 0.05). The contents of C16:0, C20:0, C18:3n6, C18:3n3, C22:0 were observed to have a significant quadratic effect with dietary protein levels (*P* < 0.05). The contents of C18:1n9c, C20:3n6, C23:0, and C22:6n3 showed significant linear and quadratic regression relationship with dietary protein levels (*P* < 0.05).

**Table 4:** The fatty acid profile (% of total fatty acids) in the muscle of grass carp fed with different dietary protein

Items	Dietary protein levels						Linear		Quadratic	
	15.91%	19.39%	22.10%	25.59%	28.53%	31.42%	*F*	*P*	*F*	*P*
C14:0	2.22 ± 0.39	2.51 ± 0.21	2.36 ± 0.12	2.41 ± 0.23	2.42 ± 0.08	2.49 ± 0.13	2.51	0.12	0.30	0.58
C14:1	0.13 ± 0.03	0.15 ± 0.01	0.13 ± 0.01	0.14 ± 0.01	0.14 ± 0.01	0.15 ± 0.01	1.31	0.26	0.48	0.50
C15:0	0.21 ± 0.02^a^	0.22 ± 0.02^ab^	0.21 ± 0.01^a^	0.23 ± 0.01^b^	0.23 ± 0.02^b^	0.25 ± 0.01^c^	28.30	< 0.01	3.03	0.10
C16:0	21.15 ± 0.34	20.86 ± 0.56	20.56 ± 0.68	20.70 ± 0.42	20.71 ± 0.38	21.21 ± 0.59	0.00	0.99	7.48	< 0.05
C16:1	11.96 ± 0.85^ab^	12.11 ± 1.00^b^	11.45 ± 0.67^ab^	11.08 ± 0.42^a^	11.31 ± 0.85^ab^	11.21 ± 0.76^ab^	6.06	< 0.05	0.65	0.43
C17:0	0.36 ± 0.03^a^	0.36 ± 0.04^a^	0.37 ± 0.03^ab^	0.40 ± 0.02^b^	0.39 ± 0.03^ab^	0.39 ± 0.02^ab^	9.42	< 0.01	0.66	0.42
C17:1	0.25 ± 0.01^ab^	0.25 ± 0.02^ab^	0.26 ± 0.02^ab^	0.27 ± 0.01^b^	0.20 ± 0.10^ab^	0.18 ± 0.12^a^	5.53	< 0.05	2.70	0.11
C18:0	3.89 ± 0.18	3.85 ± 0.30	4.01 ± 0.11	4.13 ± 0.10	4.11 ± 0.40	4.00 ± 0.29	2.67	0.11	1.29	0.26
C18:1n9t	0.35 ± 0.05^a^	0.39 ± 0.05^a^	0.37 ± 0.04^a^	0.43 ± 0.07^a^	0.41 ± 0.12^a^	0.57 ± 0.11^b^	20.27	< 0.01	3.92	0.06
C18:1n9c	32.54 ± 3.20^a^	37.21 ± 2.58^b^	37.97 ± 1.07^b^	36.94 ± 2.03^b^	36.92 ± 2.00^b^	37.12 ± 1.22^b^	8.22	< 0.01	10.03	< 0.01
C18:2n6c	9.54 ± 1.35	9.93 ± 1.09	10.58 ± 1.65	10.30 ± 0.75	10.26 ± 0.60	10.24 ± 0.90	1.22	0.23	1.34	0.26
C20:0	0.14 ± 0.02^a^	0.15 ± 0.01^ab^	0.16 ± 0.01^b^	0.16 ± 0.02^b^	0.14 ± 0.02^ab^	0.14 ± 0.01^ab^	0.03	0.87	7.90	< 0.01
C18:3n6	0.17 ± 0.02^a^	0.18 ± 0.01^ab^	0.21 ± 0.04^b^	0.20 ± 0.02^ab^	0.19 ± 0.02^ab^	0.19 ± 0.01^ab^	1.10	0.30	5.46	< 0.05
C20:1n9	0.15 ± 0.01^a^	0.17 ± 0.01^b^	0.17 ± 0.02^b^	0.16 ± 0.01^ab^	0.16 ± 0.01^ab^	0.16 ± 0.01^ab^	0.33	0.57	2.28	0.14
C18:3n3	0.97 ± 0.13^a^	1.07 ± 0.11^ab^	1.11 ± 0.14^b^	1.09 ± 0.09^ab^	1.08 ± 0.06^ab^	1.04 ± 0.06^ab^	0.82	0.37	5.58	< 0.05
C20:2	0.60 ± 0.06	0.55 ± 0.05	0.61 ± 0.10	0.57 ± 0.02	0.62 ± 0.08	0.65 ± 0.11	2.40	0.13	1.33	0.25
C22:0	1.08 ± 0.23^b^	0.89 ± 0.09^ab^	0.82 ± 0.20^a^	0.86 ± 0.18^a^	0.94 ± 0.16^ab^	0.96 ± 0.10^ab^	0.53	0.47	7.30	< 0.05
C20:3n6	0.87 ± 0.19^b^	0.63 ± 0.11^a^	0.68 ± 0.07^a^	0.67 ± 0.07^a^	0.68 ± 0.08^a^	0.65 ± 0.07^a^	7.17	< 0.05	4.99	< 0.05
C20:3n3	0.11 ± 0.03	0.12 ± 0.02	0.12 ± 0.01	0.12 ± 0.01	0.11 ± 0.04	0.11 ± 0.02	0.00	0.98	0.62	0.43
C23:0	1.96 ± 0.87^b^	1.03 ± 0.42^a^	0.98 ± 0.25^a^	1.07 ± 0.36^a^	1.12 ± 0.37^a^	1.04 ± 0.22^a^	7.10	< 0.05	6.85	< 0.05
C22:2	0.25 ± 0.04^b^	0.27 ± 0.02^b^	0.26 ± 0.03^b^	0.27 ± 0.03^b^	0.18 ± 0.11^ab^	0.15 ± 0.13^a^	8.63	< 0.01	4.14	0.05
C20:5n3	1.97 ± 0.62^b^	1.46 ± 0.29^a^	1.37 ± 0.26^a^	1.48 ± 0.25^a^	1.46 ± 0.19^a^	1.36 ± 0.12^a^	6.90	< 0.05	3.53	0.07
C22:6n3	8.73 ± 3.43^b^	5.22 ± 1.53^a^	4.84 ± 1.43^a^	5.83 ± 1.52^a^	5.82 ± 1.43^a^	5.32 ± 1.13^a^	4.82	< 0.05	5.36	< 0.05
∑SFA	31.25 ± 0.48^c^	30.14 ± 1.13^ab^	29.73 ± 0.84^a^	30.28 ± 0.57^ab^	30.30 ± 0.42^ab^	30.77 ± 0.63^bc^	0.33	0.57	12.74	< 0.01
∑UFA	68.75 ± 0.48^a^	69.86 ± 1.13^bc^	70.27 ± 0.84^c^	69.72 ± 0.57^bc^	69.70 ± 0.42^bc^	69.23 ± 0.63^ab^	0.33	0.57	12.74	< 0.01
∑MUFA	45.46 ± 3.73^a^	50.35 ± 2.85^b^	50.41 ± 1.33^b^	49.08 ± 1.86^b^	49.23 ± 1.87^b^	49.47 ± 1.74^b^	3.61	0.07	6.62	< 0.05
∑PUFA	23.26 ± 3.35^b^	19.48 ± 2.21^a^	19.82 ± 1.39^a^	20.59 ± 1.37^a^	20.44 ± 1.90^a^	19.74 ± 1.94^a^	3.65	0.07	2.84	0.10

Values are means ± SD and *n* = 6 for each group. ^a-c^ Different superscripts in the same row indicate significantly difference (*P* < 0.05)

As displayed in Table 5, the contents of Arg, Thr, Gln and Asp were markedly increased, with the protein levels rising to 22.10%, and decreased thereafter (*P* < 0.05). There was a significant quadratic regression relationship between the Arg, Thr, and Asp contents and dietary protein levels (*P* < 0.05). The maximum Pro and Met contents were observed in the 25.59% protein group and decreased thereafter. They were observed to have a highly significant quadratic regression relationship with dietary protein levels (*P* < 0.01). When the level of protein was 31.42%, the contents of Gly and Ala were notably lower than those of the other groups (*P* < 0.05). The contents of Val, Phe and Leu markedly declined as the dietary protein level increased to 22.10% and then increased gradually (*P* < 0.05). The Ile content did not change among the 15.91%–25.59% dietary protein levels and significantly increased thereafter. The content of Tyr was lower at 19.39%–22.10% dietary protein levels than that in the other groups, which was observed to have a highly significant quadratic effect with dietary protein levels (*P* < 0.01). The contents of Lys and Glu were significantly higher at the 25.59%–31.42% dietary protein levels than at the 15.91% group. The contents of Gly, Val, Ile, Leu, and Lys were observed to have a highly significant linear regression relationship with dietary protein levels (*P* < 0.01). The contents of Ala, Phe, and His were observed to have significant linear and quadratic regression relationships with dietary protein levels (*P* < 0.05). There was a highly significant quadratic regression effect between TAA, the IMP content and dietary protein levels (*P* < 0.01).

**Table 5:** The free amino acid composition (mg/100 g tissue) and IMP content (nmol/L) in the muscle of grass carp

Items	Dietary protein levels						Linear		Quadratic	
	**15.91%**	**19.39%**	**22.10%**	**25.59%**	**28.53%**	**31.42%**	***F***	***P***	***F***	***P***
Aspartate	0.20 ± 0.17^a^	0.74 ± 0.72^ab^	0.97 ± 0.43^b^	0.92 ± 0.30^b^	0.58 ± 0.28^ab^	0.51 ± 0.46^ab^	0.50	0.49	10.84	< 0.01
Glutamate	6.41 ± 1.93^a^	9.69 ± 2.93^b^	6.79 ± 1.96^ab^	7.53 ± 1.69^ab^	7.39 ± 3.90^ab^	8.38 ± 1.15^ab^	0.20	0.66	0.00	0.97
Glutamine	56.51 ± 14.78^a^	63.74 ± 10.21^ab^	73.04 ± 4.20^b^	65.89 ± 6.25^ab^	63.39 ± 17.31^ab^	65.94 ± 12.29^ab^	0.94	0.34	2.58	0.12
Glycine	189.08 ± 31.76^b^	196.48 ± 44.21^b^	131.06 ± 26.76^a^	103.88 ± 17.22^a^	101.95 ± 22.60^a^	97.33 ± 12.35^a^	65.65	< 0.01	3.47	0.07
Alanine	73.95 ± 7.62^d^	56.37 ± 7.05^c^	40.97 ± 9.41^b^	42.00 ± 8.77^b^	37.98 ± 7.82^ab^	29.00 ± 8.03^a^	100.33	< 0.01	8.42	< 0.01
Proline	253.51 ± 54.40^a^	296.52 ± 64.87^ab^	313.76 ± 48.70^ab^	336.06 ± 57.28^b^	288.66 ± 43.10^ab^	270.17 ± 45.34^ab^	0.21	0.65	8.21	< 0.01
Threonine	41.69 ± 8.24^a^	52.61 ± 9.06^ab^	60.30 ± 12.22^b^	56.40 ± 10.53^b^	51.20 ± 4.70^ab^	52.12 ± 10.69^ab^	1.82	0.19	8.09	< 0.01
Methionine	1.13 ± 0.44^a^	1.78 ± 0.66^abc^	2.38 ± 1.38^bc^	2.88 ± 0.57^c^	2.21 ± 0.82^abc^	1.68 ± 1.23^ab^	2.08	0.16	10.32	< 0.01
Valine	5.49 ± 1.54^ab^	5.59 ± 1.53^ab^	5.31 ± 0.71^a^	7.31 ± 1.22^bc^	8.61 ± 1.13^c^	8.58 ± 2.42^c^	26.06	< 0.01	1.00	0.32
Isoleucine	2.64 ± 0.45^a^	2.78 ± 0.75^a^	2.77 ± 0.39^a^	3.05 ± 0.65^a^	4.99 ± 1.24^b^	4.82 ± 1.41^b^	33.60	< 0.01	3.42	0.07
Phenylalanine	3.19 ± 0.79^b^	2.58 ± 0.71^ab^	2.32 ± 0.71^a^	4.28 ± 0.86^b^	4.41 ± 0.86^b^	4.96 ± 0.77^b^	36.88	< 0.01	6.27	< 0.05
Leucine	15.24 ± 2.48^ab^	13.24 ± 2.95^a^	12.96 ± 4.10^a^	16.74 ± 2.73^ab^	19.67 ± 3.91^b^	19.58 ± 4.51^b^	13.80	< 0.01	2.88	0.10
Tyrosine	4.48 ± 0.71^c^	2.11 ± 0.61^a^	2.21 ± 0.51^a^	2.70 ± 0.68^ab^	3.20 ± 1.04^b^	3.39 ± 0.98^b^	0.40	0.53	24.44	< 0.01
Arginine	57.46 ± 13.40^ab^	93.46 ± 33.77^b^	95.85 ± 56.11^b^	76.72 ± 32.40^ab^	57.47 ± 8.92^ab^	43.43 ± 13.02^a^	3.47	0.07	8.43	< 0.01
Serine	16.56 ± 3.54	14.19 ± 2.91	14.86 ± 5.71	13.32 ± 3.42	13.19 ± 4.42	12.53 ± 3.03	3.33	0.08	0.13	0.72
Lysine	50.46 ± 8.98^a^	55.37 ± 15.72^a^	69.94 ± 17.71^a^	104.92 ± 26.61^b^	116.01 ± 27.05^b^	154.55 ± 24.59^c^	104.12	< 0.01	3.80	0.06
Histidine	446.84 ± 67.81^a^	547.10 ± 73.00^b^	557.44 ± 49.11^b^	621.42 ± 82.94^bc^	661.77 ± 51.30^c^	566.86 ± 80.47^b^	18.45	< 0.01	11.08	< 0.01
TAA	1225.27 ± 75.89^a^	1414.85 ± 149.59^b^	1393.32 ± 146.26^b^	1466.31 ± 107.43^b^	1443.14 ± 55.46^b^	1344.20 ± 107.23^ab^	3.85	0.06	11.90	< 0.01
IMP	48.03 ± 4.82^a^	56.24 ± 3.74^bc^	57.67 ± 5.05^bc^	61.01 ± 5.48^c^	51.88 ± 5.94^ab^	51.22 ± 6.05^ab^	0.12	0.73	19.53	< 0.01

Values are means ± SD and *n* = 6 for each group. ^a-c^ Different superscripts in the same row indicate significantly difference (*P* < 0.05), Phenylalanine (*P* < 0.08)

### Histological analysis in grass carp muscle

**Fig. 1:**
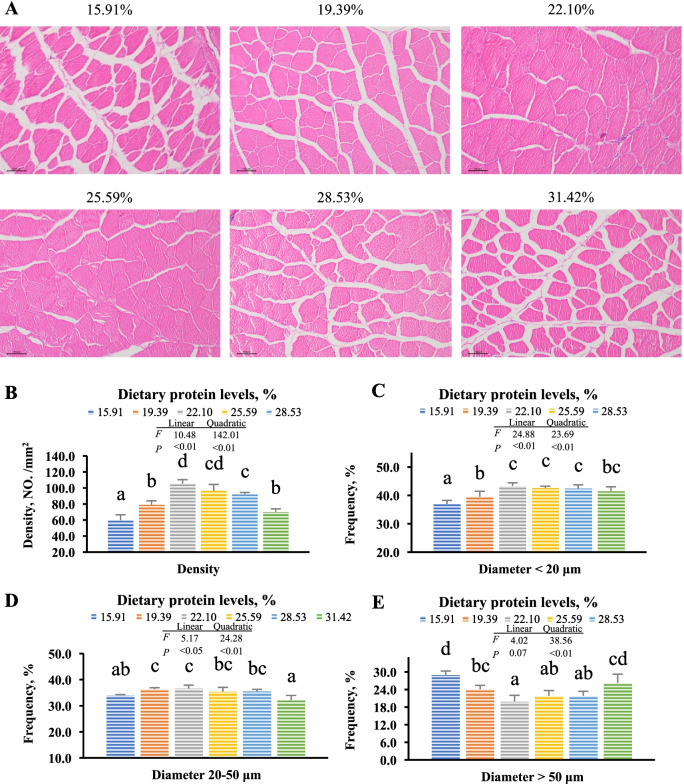
The morphology Histological analysis in grass carp muscle. **A** Transection stained by H&E

The muscle morphology is shown in Fig. 1. With the dietary protein level rising to 22.10%, the density and the frequency of muscle fibers (diameter < 20 μm and 20–50 μm) significantly increased and then decreased gradually (*P* < 0.05). These parameters were observed to have significant linear and quadratic regression effects with dietary protein levels (*P* < 0.05). The frequency of muscle fiber diameter > 50 μm declined with the dietary protein increasing to 22%, and increased thereafter. (*P* > 0.05).

### Effects of dietary protein on hardness and collagen content of muscle

As presented in Table 6, the shear force in the 19.39–22.10% protein groups were significantly higher than that in the 15.91%, 28.53% and 31.42% dietary protein groups. The collagen and HYP contents of muscle were apparently elevated when protein levels rose to 22.10% and decreased thereafter (*P* < 0.05). These parameters were observed to have significant linear and quadratic regression effects with dietary protein levels (*P* < 0.05).

**Table 6:** The shear force, Hydroxyproline, collagen and IGF-1 contents, and collagen metabolism-related enzyme activities in muscle

Items	Dietary protein levels						Linear		Quadratic	
	15.91%	19.39%	22.10%	25.59%	28.53%	31.42%	*F*	*P*	*F*	*P*
Shear force, N	2.13 ± 1.00^ab^	3.61 ± 0.66^c^	3.30 ± 0.45^c^	2.81 ± 0.54^bc^	1.86 ± 0.34^a^	2.12 ± 0.73^ab^	6.68	< 0.05	12.55	< 0.01
Hyp, μg/mg	0.31 ± 0.02^a^	0.34 ± 0.04^b^	0.60 ± 0.02^e^	0.45 ± 0.01^d^	0.46 ± 0.02^d^	0.39 ± 0.02^c^	64.14	< 0.01	288.66	< 0.01
Collagen, μg/mg	2.44 ± 0.12^a^	2.69 ± 0.31^b^	4.80 ± 0.20^e^	3.64 ± 0.11^d^	3.68 ± 0.16^d^	3.14 ± 0.19^c^	64.13	< 0.01	288.56	< 0.01
IGF-1, ng/mL	29.73 ± 2.18^a^	30.31 ± 1.74^a^	32.95 ± 1.51^ab^	33.90 ± 2.76^b^	33.57 ± 2.73^b^	30.04 ± 4.05^a^	1.87	0.18	10.87	< 0.01
LOX, ng/mL	12.76 ± 0.81^a^	13.71 ± 0.84^ab^	15.23 ± 1.43^b^	14.72 ± 1.43^b^	14.65 ± 1.62^b^	14.06 ± 1.28^ab^	4.11	0.05	8.76	< 0.01
P4H, μg/L	78.57 ± 6.39^a^	83.05 ± 6.56^ab^	88.46 ± 5.55^b^	84.95 ± 5.31^ab^	83.40 ± 7.32^ab^	83.28 ± 1.75^ab^	1.15	0.29	5.55	< 0.05
MMP2, ng/mL	88.70 ± 1.81^ab^	85.50 ± 3.18^ab^	84.22 ± 6.01^a^	88.12 ± 2.88^ab^	89.25 ± 10.21^ab^	92.37 ± 5.97^b^	2.92	0.10	3.69	0.46

Values are means ± SD and *n* = 6 for each group. ^a-e^ Different superscripts in the same row indicate significantly difference (*P* < 0.05)

### Activities of collagen metabolism-related enzymes in fish muscle

The content of IGF-1 and the activities of collagen metabolism-related enzymes in grass carp muscle are presented in Table 6. The IGF-1 content increased with increasing dietary protein (*P* < 0.05) and then decreased gradually at protein levels higher than 25.59%. The activity of P4H increased gradually when the protein levels of the diet increased from 15.91% to 22.10%, after which it showed no significant differences (*P* < 0.05). The activity of LOX was gradually enhanced within the range of 15.91%–22.10% protein (*P* < 0.05) and then showed a downward trend. There was a significant quadratic regression relationship between IGF-1, LOX, P4H and dietary protein levels (*P* < 0.05). The activity of MMP-2 gradually decreased within the range of 15.91–22.10% and then increased gradually.

### Collagen metabolism-related signaling factor mRNA and protein levels

**Fig. 2:**
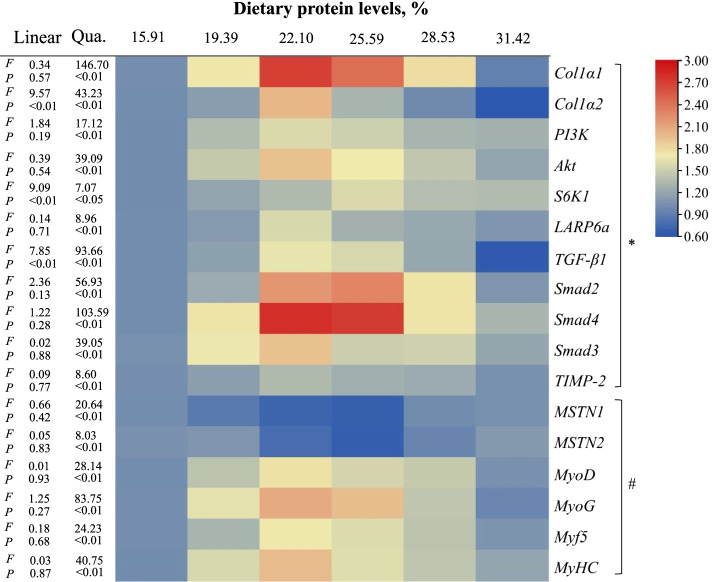
Heat map of related gene expression in muscle of grass carp. * Collagen metabolism-related signal molecules; # Factors related to muscle fibre growth. Red indicates high expression and *n* = 6 for each group

The relative expression of *Col1α1*, *Col1α2*, *PI3K*, *Akt*, *S6K1*, *LARP6a*, *TGF-β1*, *Smad2*, *Smad4*, *Smad3* and *TIMP-2* genes of muscle are exhibited in Fig. 2. The mRNA levels of *Col1α1*, *Col1α2*, *PI3K* and *Akt* were upregulated as dietary protein increased to 22.10% and then decreased at higher protein levels (*P* < 0.05). The highest mRNA levels of *LARP6a*, *TIMP2* and *S6K1* were observed in the 22.10%, 22.10% and 25.59% dietary protein level groups, respectively (*P* < 0.05), and plateaued at higher protein levels. The mRNA levels of *TGF-β1*, *Smad2* and *Smad4* were remarkably increased as dietary protein increased to 25.59%, and decreased between 28.53% and 31.42% protein of diet groups (*P* < 0.05). The *Smad3* gene expression was up-regulated when dietary protein level was increased from 15.91% to 22.10%, and then reduced gradually (*P* < 0.05). However, *MSTN1* and *MSTN2* mRNA levels were declined as dietary protein level increase from 15.91% to 25.59% (*P* < 0.05), and raised thereafter. *MyoD*, *MyoG*, *MYF5* and *MyHC* gene expressions were significantly upregulated as dietary protein levels increased and then decreased among 25.59% with 31.42% (*P* < 0.05). There was a significant quadratic regression relationship between all of these genes and dietary protein levels (*P* < 0.05). There was also a significant linear regression relationship between *Col1α2*, *S6K1*, and *TGF-β1* and dietary protein levels.

**Fig. 3:**
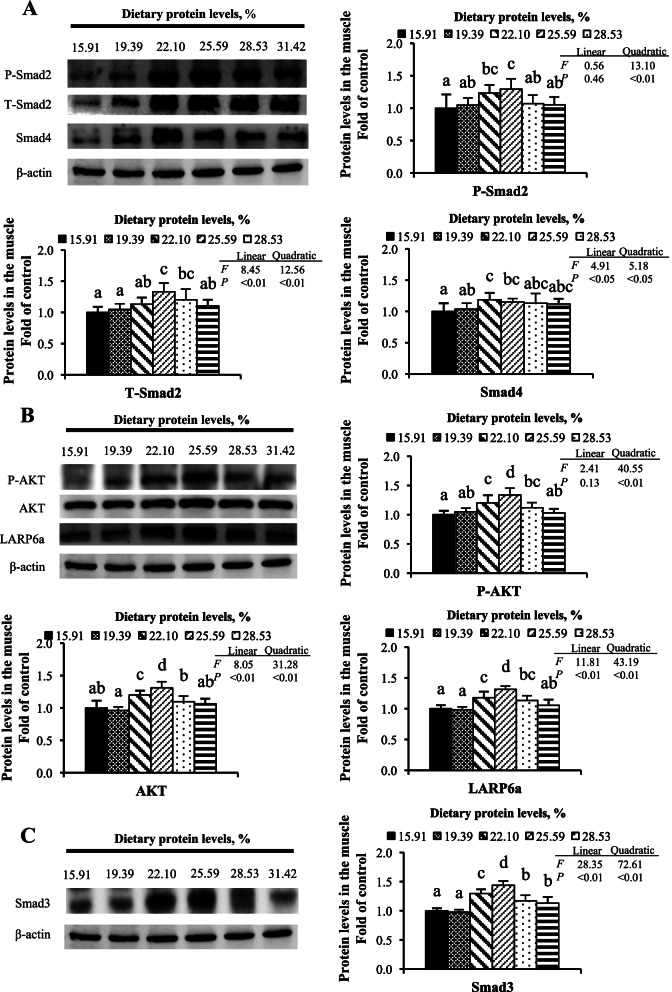
Western blot analysis of relative protein expression in the muscle of grass carp. **A** Collagen transcription related factors; **B** Related factors regulating collagen mRNA translation; **C** Collagen degradation related factors. Values are means ± SD and *n* = 6 for each group. Different letters are significantly different (*P* < 0.05)

As presented in Fig. 3, the highest Akt, p-Akt, T-Smad2, p-Smad2, LARP6 and Smad4 protein levels in muscle were observed (*P* < 0.05) when the dietary protein levels were up to 25.59%, and all declined thereafter. The Smad3 protein level of muscle was notably elevated as the increase in dietary protein rose to 22.10% and then subsequently reduced (*P* < 0.05). There was a significant quadratic regression relationship between all of these protein expression levels and dietary protein levels (*P* < 0.05). There was also a significant linear regression relationship between T-Smad2, Smad4, Akt, LARP6, and Smad3 and dietary protein levels (*P* < 0.05).

## Discussion

### Dietary protein levels improved the growth performance and chemical composition of grass carp

The present study suggested that 22.10%–25.59% of dietary protein levels increased the growth performance (FBW, PWG, SGR, and FI) of grass carp, which was consistent with that in young grass carp [16]. This finding demonstrated that the proper dietary protein levels indeed promoted the growth of grass carp. The weight gain of fish was primarily attributed to its muscle growth [41], which involved the deposition of proteins and lipids in the muscle [11]. Here, the appropriate levels of dietary protein increased the crude protein and lipid contents. We also observed that appropriate levels of dietary protein decreased ∑SFA, increased ∑UFA, ∑MUFA content, partial free amino acid contents (such as aspartate, glutamate, glycine, proline, threonine, arginine, lysine, histidine), and the IMP content. The results of this study on the reduction of the ∑SFA content were consistent with other studies [5, 36]. However, the results for unsaturated fatty acids differ from those of Montenegro et al. [30, 36], and the reason may be related to the use of different diet types and protein sources. Moreover, our results showed that the proper levels of dietary protein decreased the lactate contents and increased the cooking loss, shear force and pH value, which was consistent with Xu et al. [5]. The above results suggested that the proper levels of dietary protein improved muscle nutritional value and changed the physicochemical properties in sub-adult grass carp. However, there is no research on the mode of action of dietary protein on hardness. Therefore, we further studied the mechanism of dietary protein acting on muscle hardness.

### Dietary protein levels improved the hardness of grass carp muscle by promoting the muscle fiber growth and increasing collagen content

#### Dietary protein levels promoted the muscle fiber growth of grass carp related to MSTN and MRFs

In fish, muscle hardness is positively correlated with the density of muscle fiber [12]. We found that the proper levels of dietary protein increased the density of grass carp muscle fibers, demonstrating that proper dietary protein levels improved the growth of muscle fibers. The increase in density might be related to hyperplasia, which can be determined by the frequency of new muscle fiber diameter < 20 μm [42]. The results of this study showed that a proper level of dietary protein increased the frequency of muscle fiber diameter < 20 μm. At the molecular level, myosin heavy chain (MyHC) is a marker protein for the process of muscle fiber differentiation [43]. Our results revealed that the proper level of dietary protein upregulated the expression of the *MyHC* gene, further supporting that dietary protein indeed promoted muscle fiber growth in grass carp. Muscle fiber growth was positively regulated by MRFs (such as MyoD, Myf5, and MyoG) [44]. The present research revealed that a proper dietary protein level upregulated the mRNA expression of *MyoD*, *Myf5* and *MyoG* in muscle. Correlation analysis suggested that the density of muscle fibers was positively correlated with *MyoD* (*r* =  + 0.964, *P* = 0.002), *Myf5* (*r* =  + 0.980, *P* = 0.001) and *MyoG* (*r* =  + 0.894, *P* = 0.016) mRNA levels, indicating that the proper level of dietary protein might promote muscle fiber growth by regulating *MRFs* gene expression in grass carp muscle. Unfortunately, there are no more studies about the effect of dietary protein on MRFs, and its specific mechanism needs further research. Furthermore, muscle fiber growth was negatively regulated by MSTN. MSTN1 and MSTN2 are subtypes of MSTN [45] that inhibit proliferation and myogenic differentiation in C_2_C_12_ myoblasts [46]. Here, we first observed that *MSTN1* and *MSTN2* mRNA levels in muscle were reduced by the proper level of dietary protein, indicating that dietary protein might promote the growth of muscle fibers by reducing *MSTN* mRNA levels. However, the expression levels of *MSTN1* and *MSTN2* genes were increased at higher protein levels, which was consistent with the result in male Wistar rats (4 weeks old) [9]. The reason might be that dietary protein exceeding animal requirements enhanced the *MSTN* to prevent excessive muscle hypertrophy. These results indicated that proper levels of dietary protein probably promote muscle fiber growth by regulating *MRF* and *MSTN* gene expression in grass carp muscle.

#### Dietary protein levels increased the collagen content by regulating collagen metabolism in grass carp

In addition to the growth of muscle fibers, muscle hardness was correlated with the collagen content, which could be calculated using the Hyp content. The present results were consistent with the previous study which found that the contents of Hyp and collagen were increased in the muscle of grass carp fed optimal dietary protein levels [5]. Actually, the content of collagen depends on the metabolism of collagen (the balance between synthesis and degradation).

On the one hand, we focused on collagen synthesis. In grass carp, the main collagen in muscle was type I collagen, which consists of the α1 and α2 peptide chains (Col1α1 and Col1α2) [47], and its expression was increased along with the increase in the expression of TGF-β1, Smad2 and Smad4 [2]. Our findings revealed that optimal dietary protein levels upregulated the mRNA expression of *Col1α1*, *Col1α2*, *TGF-β1*, *Smad2*, and *Smad4* and the protein expression of p-Smad2, Smad2 and Smad4, indicating that optimal dietary protein might improve the transcription of collagen by activating the TGF-β1/Smad signaling pathway. The dietary protein regulation of TGF-β1 might be related to methionine. Experiments on grass carp have shown that methionine increased the expression of the *TGF-β1* gene in muscle [24]. In our experiment, the muscle methionine content was increased at a proper dietary protein level. After transcription, the mRNA stabilization and translation of *Col1α1* and *Col1α2* are mediated by a member of a superfamily of RNA binding proteins: LARP6, which specifically binds to the 5’ stem-loop in the *Col1α1* and *Col1α2* mRNAs [13]. We cloned the sequence of *LARP6a* in grass carp and found that the proper level of dietary protein increased the mRNA and protein expression of LARP6a, indicating that dietary protein changed the synthesis of collagen in part related to its translation. In human aortic smooth muscle cells, the expression of LARP6 and type I collagen is regulated by the endogenous growth factor IGF-1 activating the PI3K/Akt/p70S6k-signaling [18]. We subsequently demonstrated that optimal dietary protein also increased the relative expression of the *IGF-1*, *PI3K*, *Akt*, *S6K1* genes and the protein levels of Akt and p-Akt. Correlation analysis revealed that *LARP6a* mRNA levels were positively correlated with the IGF-1 content (*r* =  + 0.697, *P* = 0.124). The regulation of IGF-1 by dietary protein might be related to threonine and arginine. It has been reported that the expression of the *IGF-1* gene in the liver of hybrid catfish and blunt snout bream (*Megalobrama amblycephala*) was upregulated by threonine and arginine, respectively [10, 48]. Our results that proper dietary protein levels increased the threonine and arginine contents in grass carp muscle support the above view. The above results showed that proper dietary protein levels improved the translation of collagen, probably by increasing the expression of LARP6a via IGF-1/PI3K/Akt/p70S6K signaling.

Before collagen maturation, the peptide chains are modified by a series of modifications. P4H catalyzes the production of 4-hydroxyproline, which is crucial for the triple helix of collagen [49]. LOX catalyzes the production of peptidyl α-aminoadipic-δ-semialdehyde, which can spontaneously condense with other lysyl groups or adjacent aldehydes to cross-link [50]. Our results revealed that the proper level of dietary protein enhanced the activities of muscle P4H and LOX, indicating that dietary protein might improve the synthesis of collagen by regulating its posttranslational modification. However, there are no reports about effect dietary protein on collagen posttranslational modification, and the specific mechanism needs further research.

On the other hand, the collagen content is correlated with its degradation. MMP-2 degrades the type I collagen in common carp (*Cyprinus carpio*) muscle [20]. In zebrafish, MMP activity is inhibited by TIMP-2 [21]. TGF-β1 inhibited the activity of MMP-2 and promoted the expression of the *TIMP* gene through *Smad3* in human hepatic stellate cells [22]. This research revealed that the optimal levels of dietary protein enhanced *TIMP-2* and *Smad3* mRNA levels as well as smad3 protein levels and inhibited the activity of MMP-2. Correlation analysis revealed that MMP-2 activity was negatively related to *TIMP-2* (*r* =–0.642, *P* = 0.169) and *Smad3* (*r* = –0.794, *P* = 0.059) mRNA levels, suggesting that proper levels of dietary protein increased the collagen content. This increase is partly by decreasing collagen degradation, probably by TGF-β1/Smad3 and TIMP-2 inhibiting MMP-2 activity. No other research exists on the effects of dietary protein levels on regulating collagen degradation, and the detailed mechanism of this process needs to be studied.

### Requirements

Considering the cost of protein material and that muscle is the main output of grass carp, it is necessary to determine the protein requirement for sub-adult grass carp according to different indicators. Quadratic regression based on the SGR (*y* = –0.0021*x*^2^ + 0.1101*x*–0.4224, *P* = 0.083, *R*^2^ = 0.810) showed that the dietary protein requirement for sub-adult grass carp (721–1381 g) was 26.21%, which was lower than that for young grass carp (250 g, 28.68%) [16]. This difference might be associated with the growth stage of grass carp. The dietary protein requirement for sub-adult grass carp was determined to be 24.85% based on the collagen content through quadratic regression analysis (*y* = –0.0221*x*^2^ + 1.0983*x*–9.5742, *P* = 0.276, *R*^2^ = 0.576), which was slightly lower than the value determined based on growth performance.

## Conclusions

In conclusion, our study suggested that the optimal levels of dietary protein (22.10%–25.59%) improved the growth performance, nutritional value and hardness of grass carp muscle. We first observed that increased muscle hardness was probably associated with an increase in new muscle fiber regulated by *MRFs* and *MSTN*, as well as an increase in collagen content. Furthermore, increased muscle hardness probably be associated with increased collagen synthesis regulated by the TGF-β1/Smad2/4 signaling pathway, IGF-1/PI3K/Akt/p70S6K/LARP6a pathway, and related enzymes (P4H and LOX). In addition, increased muscle hardness is probably associated with decreased collagen degradation regulated by the TGF-β1/Smad3/TIMP-2/MMP-2 signaling pathway. Finally, the dietary protein requirements for sub-adult grass carp were determined to be 26.21% and 24.85%, according to quadratic regression of the SGR and the collagen content, respectively.

## Supplementary Information

**Additional file 1:** **Table S1.** The primer sequences and accession numbers for studied genes.

## Data Availability

The datasets are included in this article and available from the corresponding author on reasonable request.

## Abbreviations

MRFs: Myogenic regulatory factors
MyoD: Myogenic differentiation 1
Myf-5: Myogenic factor 5
MyoG: Myogenin
MSTN: Myostatin
MyHC: Myosin heavy chain
TGF-β1: Transforming growth factor-beta 1
Smad: Mothers against decapentaplegic homolog
IGF-1: Insulin-like growth factors-1
PI3K: Phosphatidylinositol-4,5-bisphosphate 3-kinase
AKT: Serine/Threonine kinase
S6K1/P70S6K: Ribosomal protein S6 kinase 1
Col1A1: Collagen type I alpha 1
Col1A2: Collagen type I alpha 2
LARP6: La ribonucleoprotein domain family member 6
TIMP2: Tissue inhibitor of metalloproteinase 2
P4H: Prolyl 4-hydroxylases
LOX: Lysyloxidase
MMP-2: Matrix metalloproteinases-2
HYP: Hydroxyproline
IMP: 5’-Inosinic acid
SGR: Specific growth rate
PWG: Percentage weight gain
