# Supplementary material for: Dietary protein levels changed the hardness of muscle by acting on muscle fiber growth and the metabolism of collagen in sub-adult grass carp (Ctenopharyngodon idella)
Source: J Anim Sci Biotechnol. 2022 Aug 25;13:109. doi: 10.1186/s40104-022-00747-7 (PMC9404606; doi:10.1186/s40104-022-00747-7)
Supplement: Supplementary file 1 — Additional file 1: Table S1. The primer sequences and accession numbers for studied genes. [file 40104_2022_747_MOESM1_ESM.docx]

**Additional file 1**

**Table S1** The primer sequences and accession numbers for studied genes

| **Gene** | **Primer sequence forward (5’ to 3’)** | **Primer sequence reverse (5’ to 3’)** | **Accession number** |
| --- | --- | --- | --- |
| *TGF-β1* | TTGGGACTTGTGCTCTAT | AGTTCTGCTGGGATGTTT | EU099588.1 |
| *Smad2* | GTCCTCCATCTTGCCTTTCAC | CTTCTCGCACCATTTCTCCTC | DQ912858.1 |
| *Smad4* | ATCACCGCCATCACCACTAAC | TATTCCTGGGGACACCACTCT | HQ596213.1 |
| *PI3K* | AGTCAGTGCCTGTGGCTGAG | CGTGTCCATGACCTCAGAGC | KY763989.1 |
| *AKT* | CCTGGTGATGAAGGAGCTGA | CTGTCAGAGAGCCTCCAGCA | KY763985.1 |
| *S6K1* | TGGAGGAGGTAATGGACG | ACATAAAGCAGCCTGACG | EF373673.1 |
| *LARP6a* | CTGAGGAGTGTGCCATCGTAG | TTCTTGGGAGGTTTGGTGCC | OL438919 |
| *Col1A1* | CAACAGCCGCTTCACATACA | GGCGATGTCAATAATAGGCAG | HM363526.1 |
| *Col1A2* | CAAGAACAGCATCGCCTACAT | AGATGGTTTATTCGTTCTGTATTCA | HM771241.1 |
| *Smad3* | ATTGAGCCTCCGAGCAACTAT | GAAAGATTTGGGGAACCTGTG | DQ912859.1 |
| *TIMP2* | GTGGTCCAGTGTTCCGTCAT | GCTGCTGTCGTTCCTCTTAAC | HQ153832 |
| *MSTN-1* | GCAGGAGTCACGTCTTGGCA | GAGTCCCTCCGGATTCGCTT | KM874826 |
| *MSTN-2* | GAACAGGCTCCGAACATCAGC | GGTTGCAGTCCTTCTTCTCC | KM874827 |
| *MyoG* | AGAGGAGGTTGAAGAAGGTC | GTTCCTGCTGGTTGAGAGA | JQ793897 |
| *MyoD* | CCCTTGCTTCAACACCAACG | TCTCCTCTCCCTCATGGTGG | GU218462 |
| *Myf-5* | GGAGAGCCGCCACTATGA | GCAGTCAACCATGCTTTCAG | GU290227 |
| *MyHC* | АСGСТСАТСАССАССАACCC | CAGCCTCCTCTGTGCCATCA | EU414733.1 |
